# Effect of vitamin D on cognitive decline: results from two ancillary studies of the VITAL randomized trial

**DOI:** 10.1038/s41598-021-02485-8

**Published:** 2021-12-01

**Authors:** Jae H. Kang, Chirag M. Vyas, Olivia I. Okereke, Soshiro Ogata, Michelle Albert, I.-Min Lee, Denise D’Agostino, Julie E. Buring, Nancy R. Cook, Francine Grodstein, JoAnn E. Manson

**Affiliations:** 1 Channing Division of Network Medicine, Brigham and Women’s Hospital/Harvard Medical School, 181 Longwood Ave, Boston, MA 02115 USA; 2 Department of Psychiatry, Massachusetts General Hospital/Harvard Medical School, Boston, MA USA; 3 Harvard T. H. Chan School of Public Health, Boston, MA USA; 4 Department of Preventive Medicine and Epidemiology, National Cerebral and Cardiovascular Center, Suita, Osaka Japan; 5 University of California at San Francisco School of Medicine, San Francisco, CA USA; 6 Division of Preventive Medicine, Brigham and Women’s Hospital/Harvard Medical School, Boston, MA 02215 USA; 7 Rush Alzheimer’s Disease Center, Rush University Medical Center, Chicago, IL USA

**Keywords:** Neurology, Randomized controlled trials

## Abstract

Low vitamin D levels have been associated with cognitive decline; however, few randomized trials have been conducted. In a trial, we evaluated vitamin D3 supplementation on cognitive decline. We included participants aged 60+ years (mean[SD] = 70.9[5.8] years) free of cardiovascular disease and cancer in two substudies in the VITAL 2 × 2 randomized trial of vitamin D3 (2000 IU/day of cholecalciferol) and fish oil supplements: 3424 had cognitive assessments by phone (eight neuropsychologic tests; 2.8 years follow-up) and 794 had in-person assessments (nine tests; 2.0 years follow-up). The primary, pre-specified outcome was decline over two assessments in global composite score (average z-scores of all tests); substudy-specific results were meta-analyzed. The pooled mean difference in annual rate of decline (MD) for vitamin D3 versus placebo was 0.01 (95% CI − 0.01, 0.02; p = 0.39). We observed no interaction with baseline 25-hydroxyvitamin-D levels (p-interaction = 0.84) and a significant interaction with self-reported race (p-interaction = 0.01). Among Black participants (19%), those assigned vitamin D3 versus placebo had better cognitive maintenance (MD = 0.04, 95% CI 0.01, 0.08, similar to that observed for Black participants 1.2 years apart in age). Thus, vitamin D3 (2000 IU/day cholecalciferol) supplementation was not associated with cognitive decline over 2–3 years among community-dwelling older participants but may provide modest cognitive benefits in older Black adults, although these results need confirmation.

**Trial registration** ClinicalTrials.gov; VITAL (NCT01169259), VITAL-DEP (NCT01696435) and VITAL-Cog (NCT01669915); the date the registration for the parent trial (NCT01169259) was submitted to the registry: 7/26/2010 and the date of first patient enrollment in either of the ancillary studies for cognitive function in a subset of eligible VITAL participants: 9/14/2011.

## Introduction

Vitamin D is a fat-soluble steroid hormone essential for bone and muscle health. Yet, the discovery of vitamin D’s autocrine pathways in multiple cell types has stimulated interest in its role in brain function^1–14^. Specifically, the vitamin D receptor is expressed in the cerebral cortex and hippocampus, critical for cognition and memory. In animals, vitamin D deficiency has been linked with deficits in brain development and aging^15–17^.

In humans, observational studies have implicated low vitamin D in cognitive impairment and dementia^18–20^, although the literature has been mixed^21,22^. Observational studies have used varying definitions of low vitamin D and of cognitive impairment/dementia; further, the issue of reverse causation is important, as low vitamin D concentrations may have resulted from lifestyle changes associated with incipient cognitive impairment/dementia. Three major previous randomized clinical trials on cognitive change^23–25^ have not shown benefits of vitamin D3. However, even a large trial (n = 4143) with 7.8 years of follow-up that observed no effect of vitamin D3 and calcium supplements on cognitive decline compared with placebo^24^ used a low dose of vitamin D3 (400 IU/day).

Thus, we investigated whether vitamin D3 supplementation of 2000 IU/day may delay cognitive decline over 2–3 years compared to placebo among healthy participants aged 60+ years in VITAL (*VIT*amin D and Omeg*A*-3 Tria*L*; NCT01169259)^26–28^, a randomized trial of vitamin D3 and omega-3 fatty acids in the prevention of major chronic diseases. In addition, we conducted pre-specified subgroup analyses by race and baseline blood vitamin D levels, given that supplementation may have stronger effects on subgroups with relatively lower blood vitamin D levels, including Black adults who are also at higher risk of cognitive decline^29–31^ and in whom we had observed suggestively different effects of vitamin D supplementation in the parent VITAL trial^32^.

## Results

The 3424 participants in VITAL-Cog were aged 60–91 years (mean = 71.9; SD = 5.4) at the first cognitive assessment (Table 1); 58.9% were women; 22.2% were Black participants and 49.8% had some years of post-graduate studies. The mean change over an average of 2.8 years of follow-up was − 0.25 (SD = 0.49) in those assigned to placebo and − 0.24 (SD = 0.48) in those assigned to vitamin D. In CTSC-Cog (Table 1), the 794 participants were aged 60–87 years (mean = 67.1; SD = 5.3) at the first cognitive assessment; 50.4% were women; 5.7% were Black participants and 55.5% had some post-graduate education. The mean change over a mean of 2.0 years of follow-up was 0.09 (SD = 0.39) in those assigned to placebo and 0.08 (SD = 0.40) in those assigned to vitamin D.

**Table 1:** Baseline characteristics of participants aged 60+ years in the VITAL cognitive substudy by vitamin D supplement assignment for VITAL-Cog (n = 3424) and CTSC-Cog (n = 794).

	VITAL-Cog (n = 3424)		CTSC-Cog (n = 794)	
	Vitamin D Group (n = 1710)	Placebo Group (n = 1714)	Vitamin D3 Group (n = 396)	Placebo Group (n = 398)
**Mean (SD)**				
Age at 1st interview, years^a^ (n = 2984 in VITAL-Cog; n = 776 in CTSC-Cog)	71.9 (5.4) (n = 1480)	71.8 (5.4) (n = 1504)	66.9 ± 5.2 (n = 385)	67.3 ± 5.4 (n = 391)
Age at 2nd interview, years^a^ (n = 2923 in VITAL-Cog; n = 515 in CTSC-Cog)	73.3 (5.7) (n = 1466)	73.4 (5.7) (n = 1457)	69.2 ± 5.1 (n = 254)	69.8 ± 5.6 (n = 261)
Cognitive test scores at 1st interview				
VITAL-Cog only tests				
TICS	33.9 (2.8)	34.0 (2.8)	–	–
OTMT-Part A (s)	10.7 (3.9)	10.3 (3.3)	–	–
OTMT-Part B (s)	38.0 (24.2)	37.9 (24.1)	–	–
Digit span backwards	6.7 (2.3)	6.8 (2.4)	–	–
CTSC-Cog only tests				
3MS	–	–	94.8 ± 4.9	94.9 ± 4.4
TMT-Part A (s)	–	–	29.2 ± 11.5	29.8 ± 9.4
TMT-Part B (s)	–	–	80.0 ± 42.9	82.5 ± 44.2
Vegetable naming test	–	–	15.6 ± 4.6	15.4 ± 4.5
Common tests across VITAL-Cog and CTSC-Cog				
TICS 10-word list recall-immediate	4.6 (1.7)	4.7 (1.7)	4.7 ± 1.3	4.7 ± 1.3
TICS 10-word list recall-delayed	2.7 (1.9)	2.7 (1.9)	2.0 ± 1.8	1.9 ± 1.7
EBMT-immediate	9.6 (1.7)	9.6 (1.8)	9.7 ± 1.7	9.7 ± 1.6
EBMT-delayed	9.3 (1.8)	9.3 (1.9)	9.3 ± 1.7	9.3 ± 1.7
Animal naming test	19.4 (5.5)	19.7 (5.6)	21.1 ± 5.9	20.3 ± 6.1
Global composite score	− 0.02 (0.57)	0.02 (0.57)	0.02 (0.63)	− 0.02 (0.56)
Baseline serum 25(OH)D (ng/mL)	32.2 (9.8)	32.5 (9.6)	28.0 (8.3)	29.1 (9.1)
**n (%)**				
Omega-3 assignment				
Active group	844 (49.4%)	855 (49.9%)	198 (50.0%)	198 (49.8%)
Placebo group	866 (50.6%)	859 (50.1%)	198 (50.0%)	200 (50.3%)
Sex				
Female	1011 (59.1%)	1005 (58.6%)	205 (51.8%)	195 (49.0%)
Male	699 (40.9%)	709 (41.4%)	191 (48.2%)	203 (51.0%)
Self-reported race/ethnicity				
Non-Hispanic White	1184 (71.3%)	1245 (73.8%)	341 (88.1%)	345 (89.2%)
Black	387 (23.3%)	356 (21.1%)	18 (4.7%)	26 (6.7%)
Other race/ethnicity^b^	90 (5.4%)	85 (5.0%)	28 (7.2%)	16 (4.1%)
Highest attained education				
High school or under	189 (11.1%)	183 (10.7%)	29 (7.3%)	34 (8.5%)
College	678 (39.7%)	664 (38.9%)	138 (34.9%)	152 (38.2%)
Post-graduate studies	842 (49.3%)	861 (50.4%)	228 (57.7%)	212 (53.3%)
Depression^c^				
No	1363 (82.7%)	1383 (82.9%)	324 (83.7%)	309 (79.8%)
Yes	285 (17.3%)	285 (17.1%)	63 (16.3%)	78 (20.2%)

*3MS* Modified Mini-Mental Status exam (range = 0–100)^33^; *CTSC* Clinical and Translational Science Collaborative Center for VITAL in Boston, MA; *EBMT* East Boston Memory Test (range = 0–12)^34^; *OTMT* Oral Trail Making Test (range = 0–120 s)^35,36^; *SD* standard deviation; *TICS* Telephone Interview for Cognitive Status (range = 0–41)^37^; *TMT* Trail Making Test (range = 0–150 s for part A and range = 0–300 s for part B)^38,39^.

^a^Characteristics as of randomization unless noted otherwise; for categorical variables, the percentages do not add to 100% due to rounding errors and numbers do not add to the total due to missing values, which were taken out of descriptive statistical analyses. In the VITAL-Cog, 501 completed only the baseline, 440 completed only the 2nd assessment and 2483 completed both assessments. In the CTSC-Cog, 497 completed both assessments, 279 completed only the baseline and 18 completed only the 2nd assessment.

^b“^Other race/ethnicity” includes “Non-Black/African-American Hispanic”, “Asian”, “Native Hawaiian or other Pacific Islander” or “American Indian/Alaska Native”.

^c^Depression is defined as a lifetime history of a depression diagnosis or of treatment for depression; current use of antidepressants; experiencing two or more weeks of depression in the past 2 years or scoring 10 points or higher on the Patient Health Questionnaire-8.

In VITAL-Cog, we did not observe an effect of vitamin D3 supplementation on cognitive function at the end of follow-up (mean = 2.8 years (range = 1.4–4.3 years); Table 2): the least squares mean for the global score was − 0.28 standard units (SE = 0.01) for the vitamin D3 group and − 0.26 (SE = 0.02) for the placebo group (mean difference = − 0.02, 95% CI − 0.06, 0.02). We observed no multivariable-adjusted differences in the global score annual rate of decline by assignment (Table 3; model 2, multivariable-adjusted mean difference = 0.01, 95% CI − 0.01, 0.02). Similarly, multivariable-adjusted differences in annual rates of decline were not significant for the secondary outcomes: 0.01 (95% CI − 0.01, 0.03), verbal memory composite score; 0.01 (95% CI − 0.01, 0.02), executive function/attention score and 0.03 (95% CI − 0.04, 0.09), TICS.

**Table 2:** Cognitive function at two assessments by Vitamin D supplement assignment, for VITAL-Cog participants aged 60 + years, (n = 3424) assessed by telephone and for CTSC-Cog participants aged 60 + years, (n = 794) assessed in person.

VITAL-COG (n = 3424; telephone assessments)						CTSC-COG (n = 794; in-person assessments)					
	Vitamin D Group		Placebo Group		Difference in score at each timepoint (Vitamin D -Placebo; 95% CI)^c^		Vitamin D Group		Placebo Group		Difference in score at each timepoint (Vitamin D-Placebo; 95% CI)^c^
	N	Mean (SE)^c^	N	Mean (SE)^c^			N	Mean (SE)	N	Mean (SE)	
**Primary outcome**						**Primary outcome**					
Global composite score^b^					Difference in score^c^	Global composite score^b^					Difference in score^c^
1st assessment score	14,800	− 0.04 (0.01)	1504	− 0.01 (0.01)	− 0.03 (− 0.07, 0.01)	1st assessment score^c^	385	0.02 (0.03)	391	− 0.03 (0.03)	0.05 (− 0.04, 0.13)
2nd assessment score	1466	− 0.28 (0.01)	1457	− 0.26 (0.02)	− 0.02 (− 0.06, 0.02)	2nd assessment score^c^	254	0.11 (0.03)	261	0.06 (0.03)	0.05 (− 0.04, 0.14)
**Secondary outcomes**						**Secondary outcomes**					
Verbal memory composite score^b^					Difference in score^c^	Verbal memory composite score^b^					Difference in score^c^
1st assessment score	14,800	− 0.02 (0.02)	1504	− 0.01 (0.02)	− 0.01 (− 0.06, 0.04)	1st assessment score^c^	385	0.01 (0.04)	391	− 0.02 (0.03)	0.03 (− 0.07, 0.13)
2nd assessment score	1466	− 0.01 (0.02)	1457	− 0.02 (0.02)	0.01 (− 0.04, 0.07)	2nd assessment score^c^	254	0.17 (0.04)	261	0.11 (0.04)	0.07 (− 0.05, 0.18)
Executive function/attention composite^b^ score^a^					Difference in score^c^	Executive function/attention composite^b^ score^a^					Difference in score^c^
1st assessment score	14,800	− 0.05 (0.02)	1504	0.01 (0.02)	− 0.05 (− 0.10, − 0.01)	1st assessment score^c^	385	0.03 (0.04)	391	− 0.05 (0.03)	0.08 (− 0.02, 0.18)
2nd assessment score	1466	− 0.53 (0.02)	1457	− 0.49 (0.02)	− 0.04 (− 0.08, 0.01)	2nd assessment score^c^	254	0.04 (0.04)	261	− 0.03 (0.04)	0.06 (− 0.04, 0.16)
TICS					Difference in score^c^	3MS					Difference in score^c^
1st assessment score	14,800	33.81 (0.07)	1504	33.92 (0.07)	− 0.12 (− 0.31, 0.08)	1st assessment score^c^	385	94.80 (0.25)	391	94.86 (0.22)	− 0.05 (− 0.71, 0.60)
2nd assessment score	1466	33.92 (0.07)	1457	33.97 (0.07)	− 0.05 (− 0.26, 0.16)	2nd assessment score^c^	254	95.69 (0.23)	261	95.52 (0.24)	0.17 (− 0.48, 0.82)

*3MS* Modified Mini-Mental Status exam (range = 0–100)^33^; *CI* confidence interval; *CTSC* Clinical and Translational Science Collaborative center for VITAL in Boston, MA; *TICS* Telephone Interview of Cognitive Status (range = 0–41)^37^.

^a^In the VITAL-Cog, 2483 completed both assessments, 501 completed only the baseline, 440 completed only the 2^nd^ assessment. In the CTSC-Cog, 497 completed both assessments, 279 completed only the baseline and 17 completed only the 2nd assessment.

^b^In the VITAL-Cog: global score is a composite score representing the mean of the z-scores of 8 tests: TICS (range 0–41), immediate and delayed recalls of the East Boston Memory Test, category fluency (animal naming test), delayed recall of the TICS 10-word list, oral trails making test A, oral trails making test B and digit span backwards. Verbal memory score is a composite score representing the mean of the z-scores of 4 tests: the immediate and delayed recalls of both the TICS 10-word list and the East Boston Memory Test. Executive function/attention score is a composite score representing the mean of the z-scores of 4 tests: trails making test A and B, category fluency tests (naming animals), and digit-span backwards. In the CTSC-Cog: the global score is a composite score representing the mean of the z-scores of 9 tests: 3MS, immediate and delayed recalls of the East Boston Memory Test, category fluency tests (naming animals and vegetables), the immediate and delayed recalls of a 10-word list and trail-making tests A and B. Verbal memory score was defined the same way as in VITAL-Cog. Executive function/attention score is a composite score representing the mean of the z-scores of 4 tests: trails making tests A and B, category fluency tests (naming animals and vegetables).

^c^Least squares means and standard errors and differences of least squares means and standard errors were derived from univariate models.

**Table 3:**
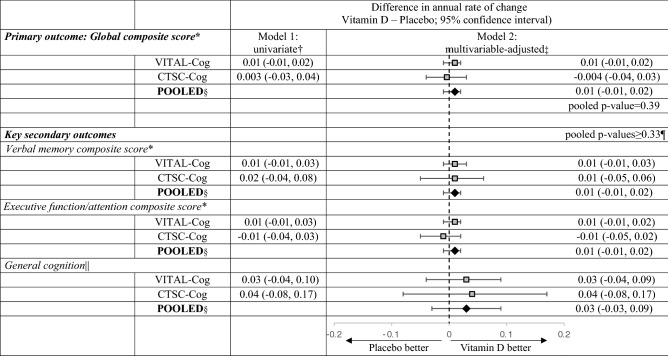
Meta-analysis of the mean differences (95% CI) in change over time among VITAL-Cog participants (n = 3424) and CTSC-Cog participants (n = 794), by Vitamin D supplement assignment.

*3MS* Modified Mini-Mental Status exam (range = 0–100)^33^, *CI* confidence interval; *CTSC* Clinical and Translational Science Collaborative center for VITAL in Boston, MA; *TICS* Telephone Interview of Cognitive Status (range = 0–41)^37^.

^a^For definitions of the global scores and the key secondary outcomes for the two populations, see footnotes for Table 2.

^b^From linear mixed models of cognitive performance: model 1 includes time since randomization modelled as a continuous variable, vitamin D assignment, and their interaction.

^c^From linear mixed models of cognitive performance: model 2 is model 1 with adjustment for 6 additional variables, omega-3 assignment (yes/no), sex (male/female), age at randomization (years), race/ethnicity (non-Hispanic white, black, other race/ethnicity), education (high school or under, college, graduate school), history of depression (yes/no; see footnote in Table 1 for definition), and the six interaction terms (products with time since randomization).

^d^Pooled using Dersimonian and Laird fixed-effects method for meta-analysis^40^ except for general cognition where the p for heterogeneity across the two substudies was 0.04 and results were meta-analyzed with random-effects.

^e^Due to the differences in scale between the TICS (0–41) used in VITAL-Cog and 3MS (range 0–100) used in CTSC-Cog, for pooling purposes, the 3MS scores were multiplied by 0.41 for conversion to the same scale as the TICS scores. As the p for heterogeneity across the two substudies was 0.04, the results were meta-analyzed with Dersimonian and Laird method incorporating random-effects^40^.

^f^None of the effects for the secondary outcomes were significant at Bonferroni-adjusted p-value of 0.0167 (= 0.05/3 secondary outcomes).

In CTSC-Cog (Table 2), we did not observe an effect of vitamin D3 supplementation on cognition at the end of follow-up (mean 2.0 years (range = 1.0–3.1 years)): the least squares mean for the global score was 0.11 (SE = 0.03) for the vitamin D3 group and 0.06 (SE = 0.03) for the placebo group (mean difference = 0.05, 95% CI − 0.04, 0.14). The multivariable-adjusted mean difference in the global score annual rate of decline was − 0.004 (95% CI − 0.04, 0.03; p = 0.83; Table 3). Similarly, multivariable-adjusted mean differences in annual rates of decline for secondary cognitive systems were not significant: 0.01 (95% CI − 0.05, 0.06) for the verbal memory composite score; − 0.01 (95% CI − 0.05, 0.02) for the executive function/attention score and 0.04 (95% CI − 0.08, 0.17) for the 3MS score (re-scaled to have range 0–41 points like the TICS). Results for individual tests are in Table S1.

We observed no heterogeneity in the results by substudy (p for heterogeneity ≥ 0.28); thus, the multivariable-adjusted results were meta-analyzed (Table 3). The pooled effect of vitamin D3 supplementation was a mean difference in the annual rate of decline of 0.01 (95% CI − 0.01, 0.02; p = 0.39) for the global score; 0.01 (95% CI − 0.01, 0.02), verbal composite score; 0.01 (95% CI − 0.01, 0.02), executive function/attention composite score; and 0.03 (95% CI − 0.03, 0.09), for general cognition (TICS/3MS).

For pre-specified interaction analysis (Table 4) by race for the global composite score, we observed a significant interaction (pooled p-interaction = 0.01), where among Black participants, the vitamin D3 group showed a significantly slower rate of decline than placebo (pooled multivariable-adjusted mean difference in annual rate of decline = 0.04, 95% CI 0.01, 0.08), but not in other races (pooled multivariable-adjusted mean difference = − 0.001, 95% CI − 0.01, 0.01). To help interpret these results, among Black participants, at the 2nd assessment in VITAL-Cog, those on vitamin D had a 0.03 standard units higher global score performance than those on placebo; this difference was equivalent to that observed with Black participants who were 1.2 years apart in age, indicating an overall modest effect. The beneficial vitamin D3 effect among Black participants was stronger for the executive function/attention score, where the effect was equivalent to the difference observed between Black participants who were 3.8 years apart in age (when the more conservative fixed effects summary was used for estimation (Table 5 footnote)). We observed no significant interaction with the other pre-specified modifier of baseline blood 25(OH)D concentrations for the global score (pooled p-interaction = 0.84; Table 4) or the secondary outcomes (pooled p-interaction ≥ 0.12; Table 5). Finally, we observed no significant interactions for the other 13 effect modifiers evaluated for the global score (pooled p-interactions ≥ 0.18; Table 4; for secondary outcomes, see Table 6).

**Table 4:**
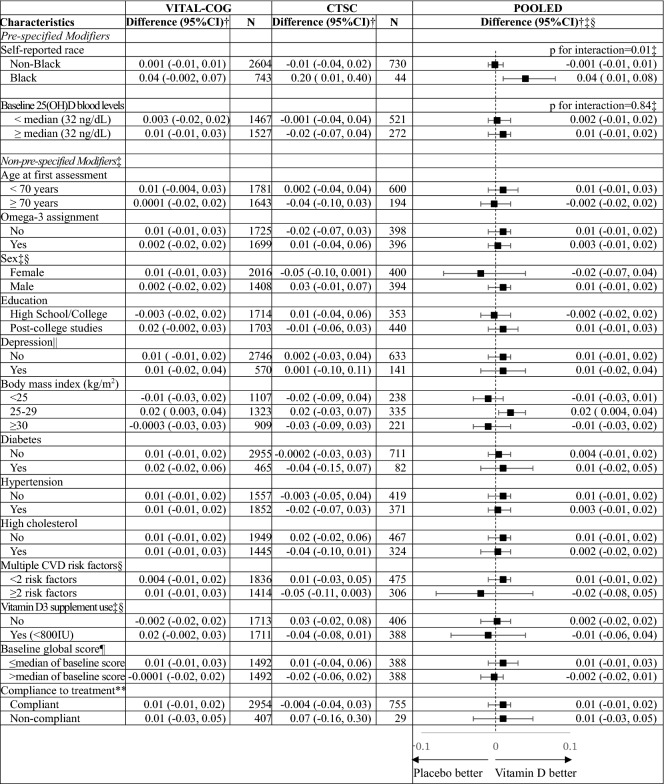
Mean difference (95% CI) in rate of change in global score between vitamin D and placebo group: effect modification by risk factors for cognitive decline.

*CI* confidence interval; *CTSC-Cog*, subset that received in-person interviews at the Harvard Clinical and Translational Science Collaborative center for VITAL in Boston, MA; *CVD* cardiovascular disease; *VITAL-Cog* subset that received telephone cognitive interviews in VITAL.

For definitions of the global scores for the two populations, see footnote for Table 2.

^a^Mean difference in annual rate of decline of vitamin D—placebo groups from multivariable-adjusted linear mixed models: see footnotes for Table 3. The stratified analyses were done among those with non-missing data on the effect modifier.

^b^Interaction terms across the two substudies were pooled using Dersimonian and Laird fixed-effects method for meta-analysis. There was significant heterogeneity (p < 0.05) for two pooled p-interactions, and for these, random effects were incorporated into the meta-analysis; the pooled p-for interaction was 0.48 for sex and 0.66 for Vitamin D supplement use (< 800 IU) outside of the trial. None of the interaction terms for the non-pre-specified modifiers were significant at the Bonferroni-adjusted p-value of 0.0038 (= 0.05/13 subgroup analyses): pooled p for interaction ≥ 0.18.

^c^Stratum-specific estimates were pooled using Dersimonian and Laird fixed-effects method for meta-analysis. There was significant heterogeneity (p-het < 0.05) for three strata, and for these, random effects were incorporated into the meta-analysis and presented in the Table. For reference, the fixed effects meta-analyzed pooled estimates were: 0.004 (95% CI − 0.01, 0.02) for females (p-het = 0.03), 0.003 (95% CI − 0.02, 0.02) for those with multiple CVD risk factors (p-het = 0.04) and 0.01 (95% CI − 0.01, 0.02) for those using Vitamin D supplements (< 800 IU) outside of the trial (p-het = 0.03).

^d^See footnote in Table 1 for definition of depression.

^e^Median for the global score was 0.05 in both the VITAL-Cog and the CTSC-Cog.

^f^Compliance is defined as taking ≥ 2/3rd of pills on all of the follow-up questionnaires between the first and the second cognitive assessment and not initiating out-of-study fish oil supplementation.

**Table 5:**
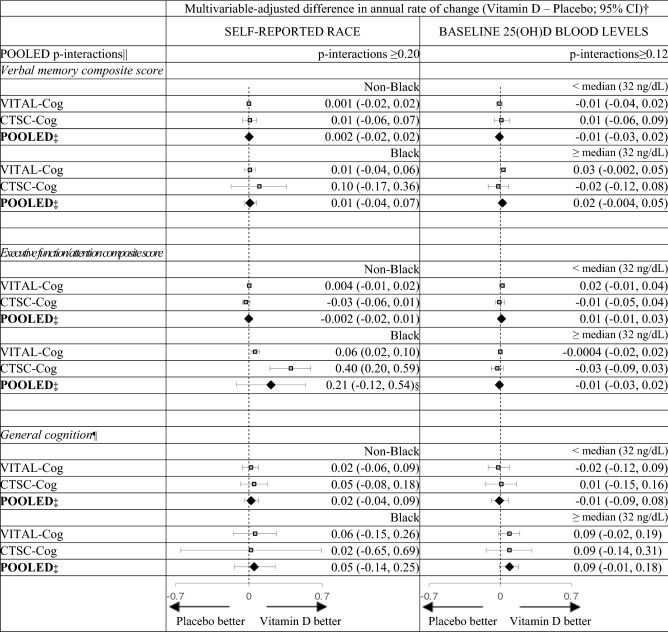
Pooled results across VITAL-Cog and CTSC-Cog for mean difference in annual rate for the secondary outcomes for vitamin D-Placebo: effect modification by race and blood 25(OH)D levels for cognitive decline.

*3MS* Modified Mini-Mental Status exam (range = 0–100)^33^; *CI* confidence interval; *CTSC* Clinical and Translational Science Collaborative center for VITAL in Boston, MA; *TICS* Telephone Interview of Cognitive Status (range = 0–41)^37^.

For definitions of the secondary outcomes for the two populations, see footnotes for Table 2.

^a^From multivariable-adjusted linear mixed models of cognitive performance (model 2) as described in footnote in Table 3.

^b^Pooled using Dersimonian and Laird fixed-effects method for meta-analysis^40^ unless otherwise noted.

^c^Pooled using Dersimonian and Laird random-effects method for meta-analysis^40^ as the p for heterogeneity was 0.001; if fixed effects methods are used, the pooled estimate was 0.07 (95% CI 0.03, 0.12).

^d^Not significant at Bonferroni-corrected p-value of 0.0167 (= 0.05/3 outcomes).

^e^Due to the differences in scale between the TICS (0–41) used in VITAL-Cog and 3MS (range 0–100) used in CTSC-Cog, for pooling purposes, the 3MS scores were multiplied by 0.41 for conversion to the same scale as the TICS scores.

**Table 6:**
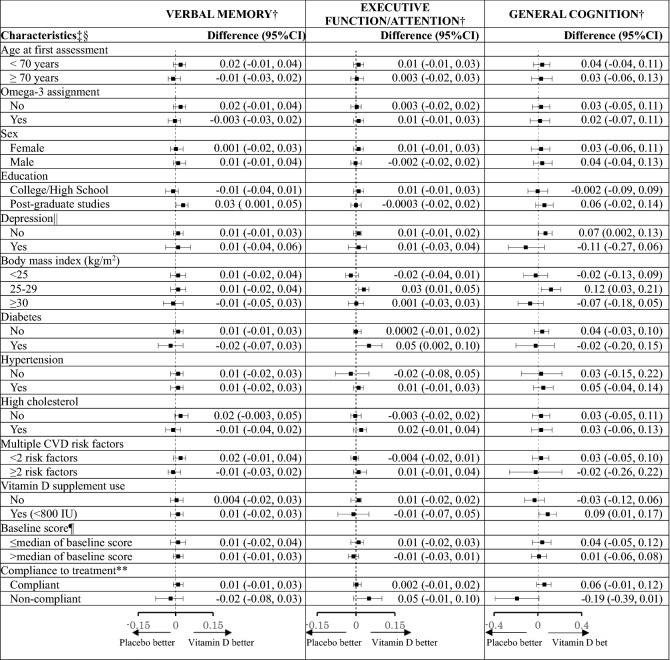
Pooled results across VITAL-Cog and CTSC-Cog for mean difference in annual rate for the secondary outcomes for vitamin D-Placebo: effect modification by risk factors for cognitive decline.

*3MS* Modified Mini-Mental Status exam; *CI* confidence interval; *CTSC-Cog* subset that received in-person interviews at the Harvard Clinical and Translational Science Collaborative center for VITAL in Boston, MA; *TICS* Telephone Interview of Cognitive Status; *VITAL-Cog* subset that received telephone interviews in VITAL.

From multivariable-adjusted linear mixed models of cognitive performance (model 2) as described in footnote in Table 3.

^a^For definitions of the verbal memory and executive function scores for the two populations, see footnotes for Table 2. For general cognition, due to the differences in scale between the TICS (0–41) and 3MS (range 0–100), for pooling purposes, the 3MS scores were multiplied by 0.41 for conversion to the same scale as the TICS scores.

^b^Interaction terms across the two substudies were pooled using Dersimonian and Laird fixed-effects method for meta-analysis. For a few interactions where there was significant heterogeneity (p < 0.05) for the estimate across the two substudies, random effects were incorporated into the meta-analysis. Among these non-pre-specified modifiers for secondary outcomes, none of the pooled p-interactions were significant at Bonferroni-adjusted p-value of 0.0038 (= 0.05/13 subgroup analyses), except for three nominally significant pooled p-interactions for education for verbal memory (p = 0.04), diabetes for executive function/attention (p = 0.04); compliance for general cognition (p = 0.01).

^c^Stratum-specific estimates were pooled using Dersimonian and Laird fixed-effects method for meta-analysis. For a few strata where there was significant heterogeneity (p < 0.05) for the estimate across the two substudies, random effects were incorporated into the meta-analysis. For reference, the fixed effects pooled estimate for executive function/attention is 0.002 (95% CI − 0.02, 0.02) for those without hypertension; 0.01 (95% CI − 0.01, 0.03) for those taking Vitamin D supplements (< 800 IU) outside of the trial; and the pooled estimate for general cognition is − 0.001 (95% CI − 0.08, 0.08) for those without hypertension and 0.03 (95% CI − 0.06, 0.13) for those with multiple CVD risk factors.

^d^For the definition of depression, see footnote in Table 1.

^e^For the verbal memory score, the median was -0.02 standard units in VITAL-Cog and 0.02 in the CTSC-Cog; for the executive memory/attention score, the median was 0.04 in VITAL-Cog and 0.02 in the CTSC-Cog; for TICS, the median was 34 in VITAL-Cog and for the 3MS in CTSC-Cog, the median was 96 (equivalent to 39 on the transformed variable to have the same range as the TICS).

^f^Compliance is defined as taking ≥ 2/3rd of pills on all of the follow-up questionnaires between the first and the second cognitive assessment and not initiating out-of-study fish oil supplementation.

In sensitivity analyses where we restricted the analyses in both substudies to those who reported no hearing impairment (68% in VITAL-Cog; 86% in CTSC-Cog; pooled mean difference in the annual rate of decline in the global score was 0.01 (95% CI − 0.01, 0.02; p = 0.37)) or restricted the analyses to those enrolled from the 1st assessment in VITAL-Cog (pooled mean difference was 0.01 (95% CI − 0.01, 0.02; p = 0.39)) or restricted the analyses in CTSC-Cog to those who did not have neuropsychiatric disorders or possible dementia at baseline (72%; pooled mean difference was 0.01 (95% CI − 0.01, 0.02; p = 0.40)) or restricted the analyses in both substudies to those who were in the top 90% of performance in each outcome (to avoid floor effects and to remove those with possible dementia, especially in VITAL-Cog; pooled mean difference was − 0.001 (95% CI − 0.01, 0.01; p = 0.93)), results were similar to the main results. Results also did not differ when we additionally adjusted for practice effects (pooled mean difference was 0.01 (95% CI − 0.01, 0.02; p = 0.31)) or when we additionally adjusted for season of cognitive assessment (pooled mean difference was 0.01 (95% CI − 0.01, 0.02; p = 0.38)).

## Discussion

In this randomized trial among generally healthy community-dwelling older participants followed for 2–3 years, supplementation with 2000 IU/day of vitamin D3 was not associated with cognitive decline. No effects were observed for the primary outcome as well as secondary outcomes of verbal memory, executive function/attention and global cognition and for both substudies where cognition was assessed by phone or in person. However, a pre-specified subgroup analysis showed cognitive benefits over time for vitamin D3 supplementation versus placebo among Black participants, but not by levels of 25(OH)D. Because the subgroup analyses by race may be due to chance, the results should be interpreted with caution and confirmed in future studies.

Randomized trials of vitamin D and cognitive decline^21,22,41^ in relatively healthy populations have shown conflicting results, with most reporting no benefit from supplementation, despite vitamin D’s potential neuroprotective anti-inflammatory and antioxidant effects^7–11,42^. In the largest (n = 4143) and longest trial (7.8 years duration) where women aged 65+ years received vitamin D3 (400 IU/day) and calcium (1000 mg/day) or placebo^24^, no association was observed between vitamin D3/calcium treatment and incident cognitive impairment or dementia, although the vitamin D3 dose was low, and was one fifth of our dose. Thus, our findings are important in suggesting that even much higher doses of vitamin D3 do not provide meaningful cognitive benefits overall. Similarly, in two European randomized trials of 2000 IU/day of vitamin D3 compared to placebo^43^ (n = 2157) or 800 IU/day^44^ (n = 273) of vitamin D3 assessed over 3^43^ and 2^44^ years among community-dwelling older persons observed no differences in change in cognitive function by intervention. Four small studies (n < 400)^23,45–47^ have evaluated even higher doses of vitamin D3 but were short-term (≤ 1 year of treatment) and also have not observed significant overall differences in cognitive change. Among Black adults, in a 3-year study among 260 older women (aged 65–73 years) where higher doses of vitamin D3 (individualized doses of ≥ 2400 IU/day needed to maintain serum 25(OH)D ≥ 30 ng/mL) and calcium (1200 mg/day) was compared to placebo and calcium (1200 mg/day), Owusu et al.^48^ observed no difference in change in MMSE performance, similar to our null finding for general cognition for Black participants; change in executive function was not assessed in this study. Thus, our study adds to the literature in that it was a long-term, large study (n > 4200) testing a relatively high dose of vitamin D3 for long durations and had a relatively large representation of Black participants.

In subgroup analyses, we observed that in Black participants, vitamin D3 supplementation was significantly associated with better cognitive maintenance in the global score and executive function/attention score. This is consistent with studies that have observed that vitamin D3 deficiency is associated most prominently with deficits in executive function^41,45,49^. While the interaction by race and baseline blood 25(OH)D levels were pre-specified, these subgroup findings were not adjusted for multiple comparisons and thus, should be interpreted with caution. We had hypothesized a priori that vitamin D3 might have particular benefits in Black participants who had lower 25(OH)D concentrations at baseline; yet, given the lack of a significant effect modification by baseline blood 25(OH)D levels in this substudy and the main trial^28^, the reasons for the specific benefits in Black participants remain unclear. A future evaluation using novel biomarkers of vitamin D (e.g., vitamin D binding protein (VDBP) or free 25(OH)D)^29,30,50,51^ and genetic variants for VDBP^52–54^ that show difference in distribution across race/ethnicity groups may be insightful. Also, it is notable that Black participants had a higher prevalence of diabetes and other cardiovascular risk factors, and lower baseline blood 25(OH)D levels, education and baseline cognitive scores, which were characteristics of subgroups for which vitamin D had a suggestively stronger beneficial effect, particularly for executive function; thus, the concentration of a multitude of risk factors for cognitive decline may have led to stronger benefits of vitamin D in Black participants.

Limitations of our study warrant consideration. First, in VITAL-Cog, cognitive assessments were conducted over the phone; however, our validation of the telephone cognitive assessment with in-person assessment showed reasonable validity, and the main results were similar in CTSC-Cog, with in-person cognitive assessments. While telephone interviews increased participation, it is possible that there was more misclassification in outcome assessment and that subtle changes were missed compared to in-person assessments. Our trial included mostly healthy, well-educated individuals (> 50% had post-graduate studies); this likely led to modest observed cognitive decline and few participants being vitamin D deficient. Both factors may have limited our ability to detect modest effects of vitamin D3 supplements on cognition. Also, while a dose of 2000 IU/day was used in the study, it is possible that the optimal dose for brain health might be higher, although the literature has been inconsistent^43,48,55^. Finally, the follow-up period of 2–3 years, with only two assessments, may have been too short to detect effects of vitamin D3 supplementation, particularly in a healthy population at relatively lower risk for cognitive decline. Although in VITAL-Cog, we did observe cognitive decline in the placebo group over 2.8 years follow-up, additional studies with longer durations of follow-up and more cognitive assessments among those at highest risk of vitamin D deficiency and cognitive decline would be important.

Our study had several strengths. This was a randomized trial including > 4200 participants, with high rates of follow-up and adherence to the assigned treatment group. In particular, there was a relatively high proportion of Black participants (19%), who are at high risk for vitamin D insufficiency^30,56–58^. Also, we were able to investigate the effect of vitamin D3 supplements on multiple cognitive domains.

In conclusion, among generally well-educated healthy adults aged 60+ years, supplementation of vitamin D3 (2000 IU/day) did not slow cognitive decline over 2–3 years, although there were modest benefits observed specifically in Black older adults that should be confirmed in future studies.

## Methods

### Study design, randomization and masking, and procedures

#### VITAL trial

VITAL^26–28^ is a completed large randomized, double-blind, placebo-controlled, 2 × 2 factorial clinical trial of vitamin D3 (vitamin D3[cholecalciferol], 2000 IU/day) and marine omega-3 fatty acid (Omacor^®^ fish oil, eicosapentaenoic acid + docosahexaenoic acid, 1 g/day) oral supplements in the primary prevention of cancer and cardiovascular disease. Participants were free of cancer (except non-melanoma skin cancer) and cardiovascular disease. Participants (n = 25,871 US men aged ≥ 50 and women aged ≥ 55 years) were randomized from 2011 to 2014 and were required to limit using out-of-study supplemental vitamin D3 to ≤ 800 IU/day, supplemental calcium to ≤ 1200 mg/day, and to avoid using omega-3 fatty acid supplements. Supplementation with 2000 IU/day vitamin D3 for one year in VITAL led to a 40% mean increase in 25-hydroxyvitamin D (25(OH)D) levels (from 29.8 to 41.8 ng/mL)^28^. The VITAL trial main phase has been completed, and its trial design (including details on randomization and masking)^26^ and main findings have been published^27,28^. The marine n-3 arm results for the cognitive substudies have been analyzed separately^59^.

### Participants

We used data from two distinct subsets of VITAL participants. Although cognitive function was not the main planned outcome to be evaluated in the parent VITAL trial, assessing cognitive function was planned before the start of the trial and baseline cognitive function assessments were planned to occur before randomization as much as possible. One subset (VITAL-Cog; NCT01669915); n = 3424) completed cognitive assessments by phone with randomization and again 2.8 years later. Another subset (CTSC-Cog; n = 794 in an ancillary study of depression (VITAL-DEP; NCT01696435)) completed in-person cognitive assessments with randomization and again 2.0 years later.

**Figure 1:**
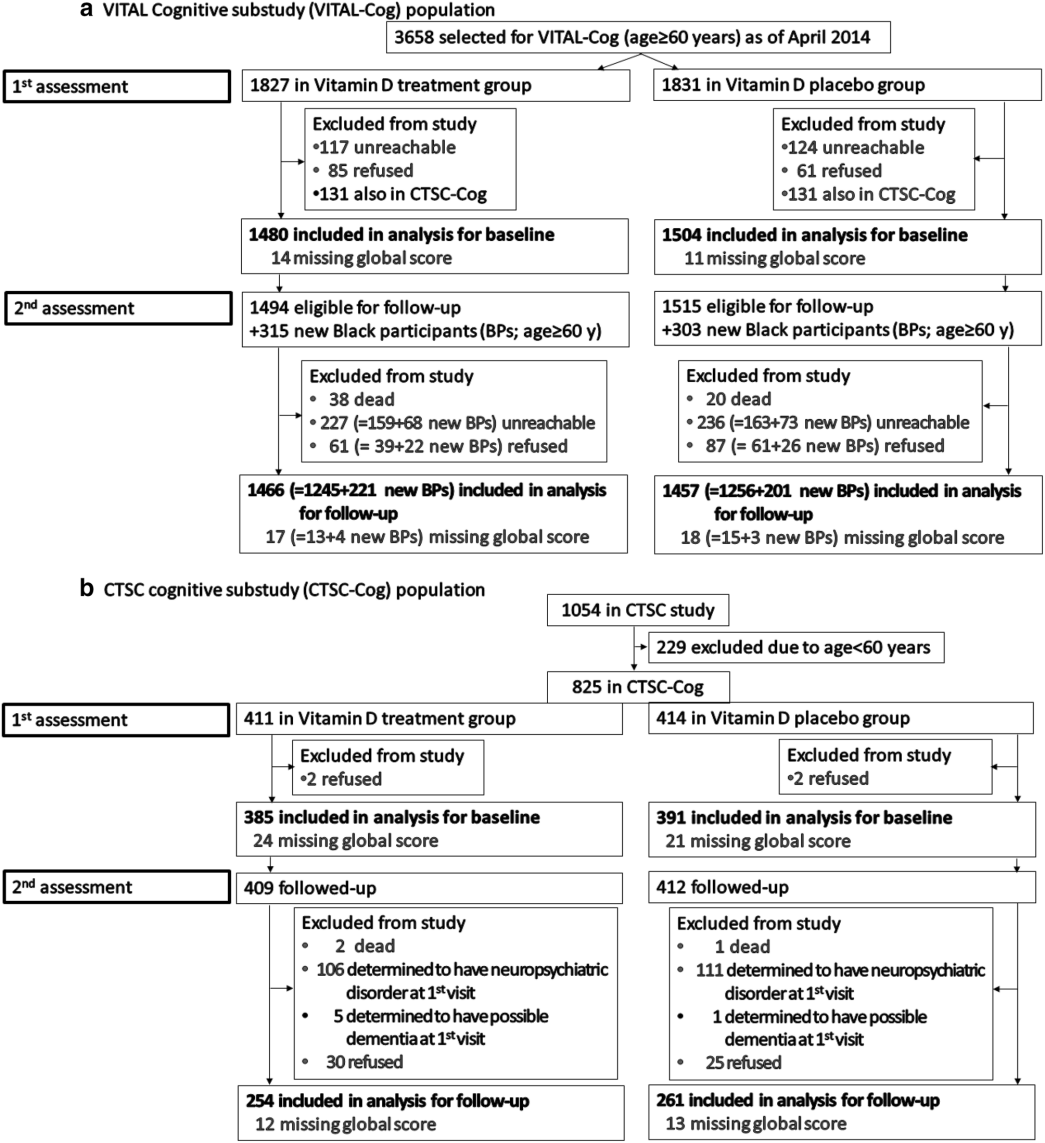
(**a**) Flow of Participants in the VITAL-Cog Ancillary Study to the VITAL Trial. (**b**) Flow of Participants in the subset of CTSC-Cog participants in the VITAL-DEP Ancillary Study to the VITAL Trial.

In VITAL-Cog, the baseline cognitive interview was conducted from September 2011 through April 2014 (mean = 1 month before randomization; range of 1.2 years before to 0.5 years after randomization (1.31% done > 1 month after randomization); Fig. 1a). Of 3658 eligible people as of April 2014 and we attempted to contact, 241 (7%) were unreachable, and of 3417 contacted, 3271 (96%) participated. We further excluded 262 participants who were also in the CTSC-Cog, leaving 3009 participants (2984, including 317 Black participants, with complete scores on all tests and 25 with scores missing on some tests). For the 2nd cognitive assessment (February 2013 to June 2016), of the 3009 who participated in the 1st assessment, 58 died (2%) and 322 were unreachable (11%). Of the 2629 contacted, 100 (4%) refused, and 2529 (96%) participated (2501 with complete scores on all tests and 28 with scores missing on some tests).

To allow for enough time for follow-up assessments within the trial period and because we had reached the target of 3000 participants, we stopped administering baseline cognitive assessments in April 2014, even though there were additional eligible participants. However, to increase the number of Black participants, at the initiation of 2nd assessments, we invited 618 additional eligible Black participants (Fig. 1a: aged 60+ years at randomization and willing to be part of VITAL-Cog). Of 618 Black participants, 141 (23%) could not be contacted. Of 477 contacted, 48 refused (10%) and 429 (90%) participated (November 2014 to June 2016; 422 with complete scores on all tests; and 7 with scores missing on some tests). Thus, the total number of unique individuals in VITAL-Cog was 3424: 2984 with complete baseline assessments and 2923 (= 2501 + 422 new Black participants) with complete follow-up assessments.

In CTSC-Cog, the baseline assessment occurred from January 2012 to March 2014 (mean = 0.5 month before randomization; range of 3.0 months before to within 1 month after randomization). For CTSC-Cog (Fig. 1b), we excluded 229 participants aged < 60 years and four people who refused participation, leaving 821 participants (776 with complete scores on all tests and 45 with scores missing on some tests). A 2-year follow-up in-person interview was conducted from January 2014 through April 2016. Of 821 who participated in the baseline assessment, 3 died (0.4%), 217 (26%) were ineligible for VITAL-DEP due to their baseline assessments showing neuropsychiatric disorders, 6 (1%) showed neuropsychiatric disorders and possible dementia, 55 (7%) refused; and 540 participated (515 with complete scores on all tests and 25 with scores missing on some tests). The total number of unique individuals in CTSC-Cog was 794 (including 44 Black participants): 776 with complete baseline assessments and 515 with complete follow-up assessments.

### Standard protocol approvals, registrations, and patient consents

The research followed the Declaration of Helsinki, and this substudy protocol was approved by the institutional review board of the Brigham and Women’s Hospital. Written informed consent was obtained directly from VITAL participants and CTSC-Cog participants or from their legally authorized representatives/next of kin^26^; for VITAL-Cog, completion of cognitive tests was considered as implied consent.

### Participants and outcomes: VITAL-Cog study population and telephone cognitive function assessments

In VITAL-Cog substudy, the eligibility criteria were age 60+ years and in the screening questionnaire, being willing to participate in cognitive function assessments. Cognition was assessed by telephone by trained interviewers, with eight neuropsychological tests assessing general cognition (Telephone Interview of Cognitive status (TICS; range = 0–41 points)), verbal memory, and executive function/attention (see details in the “Supplementary Methods”). We derived a global composite score by averaging the z-scores of the eight individual tests (based on the baseline test distributions). When generating composite scores for the 2nd assessment, the baseline means and SDs of scores from VITAL-Cog were used. Our primary, pre-specified outcome was the annual rate of change of the global composite score, and for secondary outcomes, we also evaluated the TICS and composite scores for verbal memory and executive function/attention (“Supplementary Methods”).

### Participants and outcomes: CTSC-Cog study population and in-person cognitive function assessments

A subgroup of 1054 VITAL participants received in-person health assessments, including cognitive assessments as part of VITAL-DEP^60^, by trained interviewers at the CTSC in Boston with randomization (CTSC-Cog). The in-person cognitive battery included nine cognitive tests assessing general cognition (Modified Mini-Mental State (3MS; range = 0–100)^33^), verbal memory and executive function/attention. The CTSC global composite score, the primary outcome, was calculated as the average of the z-scores for the nine assessments, using the CTSC-Cog baseline means and SDs, for both baseline and follow-up; secondary outcomes included the 3MS and verbal memory and executive function/attention composite scores (“Supplementary Methods”).

### Validation study of the VITAL-Cog telephone cognitive assessment

Cognitive assessment by phone has been extensively validated^61,62^. In VITAL-Cog, we validated our telephone cognitive assessment against in-person assessments among a subset of 181 of the 262 CTSC participants with both assessments who had the two within 1 month of each other. We compared the global composite score derived from scores on the eight tests administered by telephone versus a similar score derived from the nine tests administered in-person. The intraclass correlation between the two modes was 0.64, supporting the validity of our telephone cognitive interview (“Supplementary Methods”).

### Statistical analyses

We compared characteristics at randomization by treatment group using Wilcoxon’s rank-sum tests for continuous variables and chi-square tests for proportions. Primary analyses were conducted using the intention to treat principle. For each substudy, linear mixed-effects models with random intercepts were used to estimate the mean change in participants’ scores as a function of time (years between randomization and each assessment), treatment assignment, and their interaction^63^. We fitted models by maximum likelihood, incorporating the longitudinal correlation within participants (using unstructured covariance structure); for statistical testing, we used Wald tests. We calculated multivariable-adjusted mean differences in annual rate of decline and 95% confidence intervals (CIs); information on covariates at pre-randomization were collected by questionnaires. We used two models: model 1 included just the treatment group, while model 2 was additionally adjusted for age at randomization (years), sex, highest attained education, race, omega-3 treatment arm assignment, and depression history.

In secondary analyses, we evaluated potential effect modification by race and baseline blood vitamin D levels, which were pre-specified given that supplementation may have stronger effects on subgroups with relatively lower blood vitamin D levels such as Black participants^29,30^. We also evaluated effect modification by testing the 3-way interaction terms in multivariable-adjusted linear mixed models for 13 possible risk factors of cognitive decline (based on self-report on pre-randomization questionnaires): age, sex, omega-3 fatty acid assignment, education, depression, body mass index, diabetes, hypertension, high cholesterol, multiple CVD risk factors, out-of-study vitamin D3 supplement use, baseline score and compliance (over the entire follow-up period).

For the primary outcome of global score and for the two pre-specified subgroup analyses, the significance tests were 2-sided, and the significance level was p-value < 0.05. For the secondary outcomes and subgroup analyses, multiple comparisons were adjusted using Bonferroni corrections.

We first evaluated associations separately by substudy and then pooled the substudy-specific results using the Dersimonian and Laird meta-analytic approach incorporating fixed-effects^40^. Because the TICS and 3MS had different scales, for pooling, we multiplied the 3MS scores by 0.41 to generate the same scale as the TICS.

In sensitivity analyses, we restricted the analyses in both substudies to those who reported no hearing impairment (68% in VITAL-Cog; 86% in CTSC-Cog), restricted the analyses to those enrolled from the 1st assessment in VITAL-Cog (to ascertain whether missingness in the data can be assumed to be missing at random), restricted the analyses in CTSC-Cog to those who did not have neuropsychiatric disorders or possible dementia at baseline (72%), and restricted the analyses in both substudies to those who were in the top 90% of performance in each outcome. In additional analyses, we additionally adjusted for practice effects by adjusting for the number of prior assessments and in alternate models, we additionally adjusted for the season of cognitive assessment as vitamin D levels may depend on season of the year.

For statistical analyses, we used SAS (SAS release 9.4; SAS Institute Inc, Cary, NC). For the cognitive ancillary substudies, there was no data monitoring committee. This study is registered with ClinicalTrials.gov VITAL-Cog (NCT01669915), VITAL-DEP (NCT01696435) and VITAL (NCT01169259).

## Supplementary Information

Supplementary Information.

## Data Availability

The corresponding author can be contacted for de-identified data requests. Analysis proposal requests will require review and approval by the VITAL Publications & Presentations Committee and appropriate IRB approval. Once approved and a data access agreement has been executed, deidentified data generated from this research will be made available to affiliated investigators through secure databases for the prespecified analysis.
