# Supplementary material for: Effect of vitamin D on cognitive decline: results from two ancillary studies of the VITAL randomized trial
Source: Sci Rep. 2021 Dec 1;11:23253. doi: 10.1038/s41598-021-02485-8 (PMC8636504; doi:10.1038/s41598-021-02485-8)
Supplement: Supplementary file 1 — Supplementary Information. [file 41598_2021_2485_MOESM1_ESM.pdf]

SUPPLEMENTARY APPENDIX

Table of Contents

Members of the VITAL Research Group 3
Supplementary Methods 4
eMethods 1. VITAL-Cog Telephone Cognitive Function Assessment 4
eMethods 2. VITAL-Cog Secondary Cognitive Outcomes 4
eMethods 3. CTSC Study Population 4
eMethods 4. CTSC Cognitive Assessment 4
eMethods 5. CTSC Outcome Derivation 4
eMethods 6. Validation study of the VITAL-Cog telephone cognitive assessment 5
Supplementary Table S1 7

Members of the VITAL Research Group

VITAL Steering Committee:

JoAnn E. Manson (Chair), Julie E. Buring (Chair), Nancy R. Cook, I-Min Lee, William Christen, Shari S. Bassuk, Samia Mora, Heike Gibson, David Gordon, Trisha Copeland, Denise D’Agostino, Georgina Friedenberg, Claire Ridge, Vadim Bubes, Edward L. Giovannucci, Walter C. Willett (all at Brigham and Women’s Hospital, Harvard Medical School, Boston; Drs. Manson, Buring, Cook, Lee, Giovannucci and Willett are also at the Harvard T.H. Chan School of Public Health).

Scientific consultants:

John Baron (University of North Carolina, Chapel Hill),
Michael Holick (Boston Medical Center), Bruce Hollis (University of South Carolina).

Other Members of the VITAL Research Group:

(Brigham and Women’s Hospital): Christine M. Albert, Diane Gold, Meryl LeBoff, Olivia Okereke, Aruna Pradhan, Howard Sesso, Wendy Chen, Paulette Chandler, J. Michael Gaziano, Olga Demler, Kathryn Rexrode, Karen Costenbader, John Forman, Erik Alexander, Sonia Friedman, Jeffrey Katz, Shumin Zhang, Jennifer Lin, Joseph Walter, Julie Duszlak, Kate Kalan, Jean MacFadyen, Natalya Gomelskaya, David Bates, Ara Sarkissian, Mary Breen, Yeulolani Andrade, Manickavasagar Vinayagamoorthy, Chunying Li, Eunjung Kim, Franco Giulianini, Gregory Kotler, Marty Van Denburgh, Rimma Dushkes, Yanyan Liu, Eduardo Pereira, Lisa Fields-Johnson, George Menjin, Lucy Liu, Lauren Girard, Scott Zeller, Naomi Riches, Katelyn Hasson, Ellen Bhang, Maria Revilla, Elena McCarthy, Alex Moran, Kristen Haise, Leah Arsenault, Philomena Quinn, Sancia Grimes, Ivan Fitchorov, Kurt Schwerin, Shamikhah Curry, Annie Murray, Angela Zhang, Diana Walrond-Williams, Alison Weinberg, Chris Pfeffer, Margarette Haubourg, Viviane Nguyen, Henry Ouellette, Rolando Rodriguez, Tony Montgomery, Keith Morse, Vincent Guzman, Megan Perry, Sandra Weekes, Doug Smith, Allison Clar, Sara Curran, Yaneve Fonge, David Hibbert, Louisa Paine, Kelly Royce, Courtney Splaine, Jennifer McMahon, David Eldridge, Laura Hand, Kay Inandan, Meghan Rieu Werden, Harriet Samuelson, Andrea Hrbek, Megan Mele, Eileen Bowes, Mary Anne Ryan

(Massachusetts General Hospital, Boston): Carlos Camargo, Jacqueline Danik, Ravi Thadhani

(Vanderbilt University, Nashville): Thomas Wang

(Rush University Medical Center, Chicago): Raj C. Shah

(University of California, San Francisco): Michelle A. Albert

(Emory University): Carlos Kase

(Centers for Disease Control and Prevention, Vitamin D Standardization Program): Hubert Vesper and Julianne Botelho.

Data and Safety Monitoring Board

(Voting Members): Lawrence S. Cohen, MD; Theodore Colton, ScD; Mark A. Espeland, PhD; Craig Henderson, MD; Alice H. Lichtenstein, ScD; Rebecca A. Silliman, MD, PhD; and Nanette Wenger, MD (Chair). Ex-officio members include Josephine Boyington, PhD, MPH; Rebecca Costello, PhD; Cindy Davis, PhD; Peter Greenwald, MD; Gabriela Riscuta, MD; and Harold Seifried, PhD.

Supplementary Methods

eMethods 1. VITAL-Cog Telephone Cognitive Function Assessment

General cognition was assessed with the Telephone Interview of Cognitive status (TICS; range=0-41 points), a telephone adaptation of the Mini-Mental State Exam (MMSE).[1] Verbal memory was evaluated with 4 tasks: the immediate and delayed recalls of both the East Boston Memory Test (EBMT; range=0-12)[2] and the TICS 10-word list (range=0-10).[1] To assess executive function/attention, a test of category fluency was administered where participants were asked to name as many animals as possible in one minute.[3] We also administered an assessment of attention/processing speed, the digit span backwards test (range=0-12 points), which asked participants to repeat backwards a series of digits,[4] and the Oral Trail Making Test (TMT), Part A (range=0-120 seconds), which asked participants to count from 1 to 25 as fast as possible. For assessing executive function, the Oral TMT Part B (range=0-120 seconds) was given, which asked participants to count to 13 and state the alphabet in alternating order (1-A, 2-B, and so on until 13-M).[5,6]

eMethods 2. VITAL-Cog Secondary Cognitive Outcomes

The primary outcome of interest was the global composite score. Secondarily, we examined verbal memory and executive function/attention, as these most strongly predict the risk of Alzheimer's disease and vascular dementia, respectively.[7,8] We derived the verbal memory composite score by averaging the z-scores of 4 tests: the immediate and delayed recalls of both the EBMT and the TICS 10-word list. We calculated an executive function/attention composite score by averaging the z-scores of 4 tests: the category fluency test,[9] the trail making tests A and B, and the digit span backwards test. Due to the skewness of the distribution of the trail making tests, we applied a square root transform and then calculated z-scores.

eMethods 3. CTSC Study Population

A sub-group of 1054 VITAL participants received in-person detailed health assessments at a CTSC site in Boston before randomization.[10] Health assessments included medical history and physical exam, with measurement of height, weight, other anthropometric indices, and blood pressure as well as various measures for ancillary studies, including cognitive assessments. All participants provided informed consent for the CTSC evaluation.

Because the CTSC cognitive assessments over time were part of VITAL-DEP (NCT01696435), which was a study of late-life depression, those ineligible for the 2nd in-person CTSC were the following, determined as of baseline: 1) diagnosis for any depressive disorder, alcohol/substance abuse or dependence in the past 12 months, psychotic disorder, bipolar disorder, OCD or PTSD; 2) cognitive impairment in the dementia range; 3) any factor that can significantly impact patient safety, ability to perform the testing procedures or compliance (e.g., transportation problems resulting in transit time of >3 hours to or from the CTSC); 4) unstable symptoms (e.g., suicidality, homicidality, mania, psychosis).

eMethods 4. CTSC Cognitive Assessment

Cognitive function was assessed in-person by trained interviewers, using nine cognitive tests assessing general cognition (Modified Mini-Mental State (3MS; range=0-100),[11] verbal memory and executive function / attention. As per the protocol for VITAL-DEP (NCT01696435), first, the Hearing Handicap Inventory Screening Version (HHIE-S) was administered to all participants. Those who scored at >50% likelihood of significant hearing impairment were administered the Modified Mini-Mental State (3MS) (range=0-100) and not the other cognitive tests. General cognition was assessed by the 3MS.[11] Verbal memory was assessed with the same 4 tests used in VITAL-Cog. To partially assess executive function/attention, two tests of category fluency were also administered (the animal naming test and a test where participants were asked to name as many vegetables as possible)[3] along with the TMT part A (range= 0-150 seconds), which asked participants to draw lines to connect the numbers from 1 to 25 as fast as possible, and the TMT part B (range= 0-300 seconds), which asked participants to draw lines to connect numbers (1 – 13) and the alphabet (A – L) in alternating order (1-A, 2-B, and so on until 13-M) as fast as possible. For the primary outcome, we evaluated a global composite score with all 9 measures and derived similar measures for secondary outcomes as in VITAL-Cog.

eMethods 5. CTSC Outcome Derivation

Similar to VITAL-Cog, the primary outcome of interest was the global composite score, which, in the CTSC study population was derived by averaging the standardized scores of all 9 tests, using the baseline distributions in the CTSC as the standard. Secondarily, we were interested in the outcomes of 3MS, verbal memory and executive

function/attention. We calculated the verbal memory composite score by averaging the z-scores of 4 tests: the immediate and delayed recalls of both the EBMT and a 10-word list. We derived the executive function/attention composite score by averaging the z-scores of 4 tests: the 2 category fluency tests for animal naming and vegetable naming,[9] and the trail making tests A and B (square root transformed values for both). At the two-year follow up, we similarly calculated the composite scores for global, verbal memory, and executive function/attention for the 2nd assessment, by using means and SDs of the baseline CTSC-Cog scores. To pool the results of the 3MS in CTSC-Cog with the TICS in VITAL-Cog, a scaling factor of 0.41 was applied to the 3MS scores before analyses were conducted.

eMethods 6. Validation study of the VITAL-Cog telephone cognitive assessment

In VITAL-Cog, from December 2011 to April 2014, we validated our telephone cognitive assessment against in-person assessments in 181 CTSC participants. Roughly half (n=93) had their telephone cognitive interview first then the in-person evaluation within 1 month; while the other half (n=88) were first assessed in-person then by telephone, also within 1 month.

We compared the global composite score derived from scores on all the 8 tests administered by telephone and that derived from scores on all the 9 tests administered in-person. The intraclass correlation between the two modes for the global composite score in those who completed the assessment by telephone first and then in-person was 0.76 (Spearman correlation=0.71, p<0.0001) and in those who completed the in-person assessment followed by the telephone interview the intraclass correlation was 0.52 (Spearman correlation=0.59, p<0.0001). Thus, these results, similar to those of other studies,[12,13] support the validity of our telephone cognitive interview compared to in-person assessments. Findings on the individual tests showed similar correlations. A Bland-Altman plot[14] showed that the mean bias ± SD between the two global scores was -0.09 ± 0.47, and the limits of agreement were -1.01 to 0.83 (Supplementary Fig. S1); in general, the plot indicates that the points are scattered above and below the zero line such that there is no bias of one approach versus the other.

Supplementary Figure S1. Bland-Altman Plot

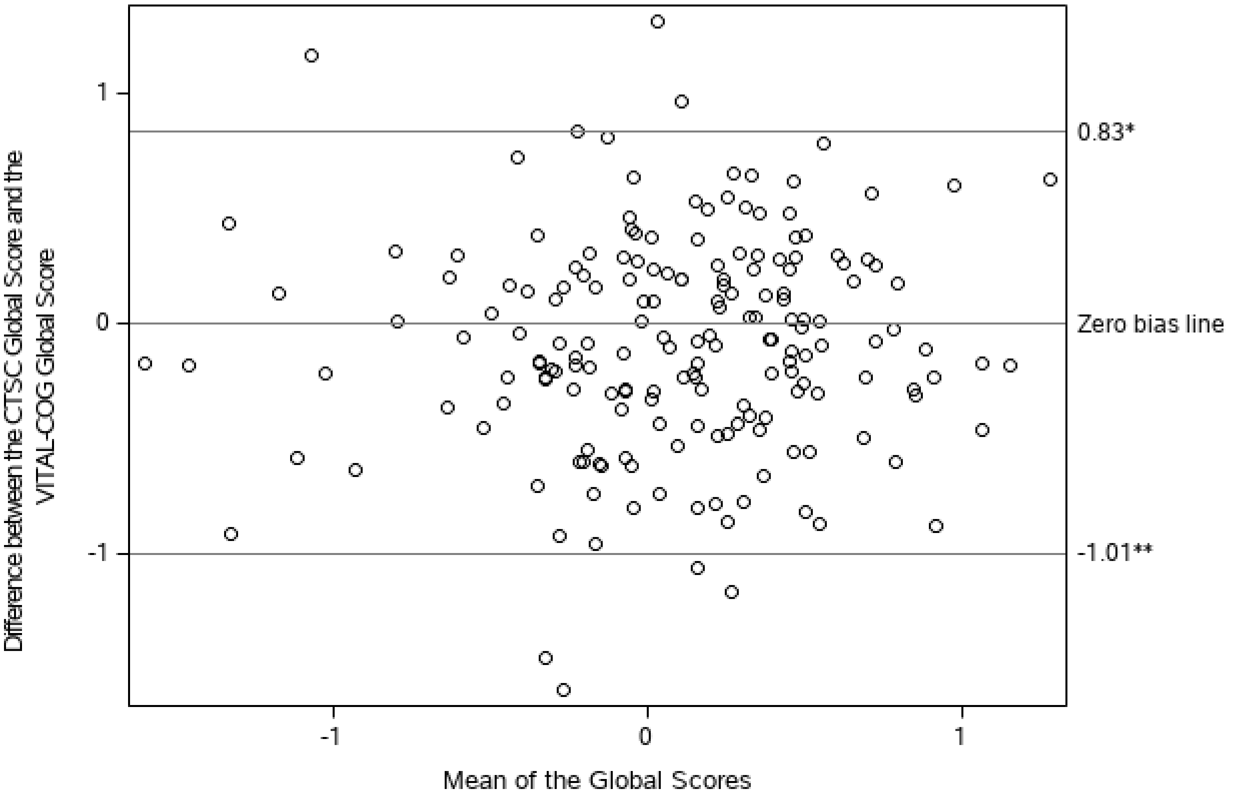

*: 95% CI Upper Limit
**: 95% CI Lower Limit

REFERENCES

1 Brandt, J. & Folstein, M. F. *Telephone Interview for Cognitive Status: Professional Manual.* (Psychological Assessment Resources, Inc, 2003).

2 Albert, M. *et al.* Use of brief cognitive tests to identify individuals in the community with clinically diagnosed Alzheimer's disease. *Int J Neurosci* **57**, 167-178 (1991).

3 Monsch, A. U. *et al.* Comparisons of verbal fluency tasks in the detection of dementia of the Alzheimer type. *Arch Neurol* **49**, 1253-1258, doi:10.1001/archneur.1992.00530360051017 (1992).

4 Wechsler, D. *WMS-R: Wechsler Memory Scale-Revised: Manual.* (Psychological Corporation, 1987).

5 Abraham, E., Axelrod, B. N. & Ricker, J. H. Application of the Oral Trail Making Test to a mixed clinical sample. *Arch Clin Neuropsychology* **11**, 697-701 (1996).

6 Bastug, G. *et al.* Oral trail making task as a discriminative tool for different levels of cognitive impairment and normal aging. *Arch Clin Neuropsychol* **28**, 411-417, doi:10.1093/arclin/act035 (2013).

7 Looi, J. C. & Sachdev, P. S. Differentiation of vascular dementia from AD on neuropsychological tests. *Neurology* **53**, 670-678, doi:10.1212/wnl.53.4.670 (1999).

8 Reed, B. R. *et al.* Profiles of neuropsychological impairment in autopsy-defined Alzheimer's disease and cerebrovascular disease. *Brain* **130**, 731-739, doi:10.1093/brain/awl385 (2007).

9 Jacobs, D. M. *et al.* Development of a novel cognitive composite outcome to assess therapeutic effects of exercise in the EXERT trial for adults with MCI: The ADAS-Cog-Exec. *Alzheimers Dement (N Y)* **6**, e12059, doi:10.1002/trc2.12059 (2020).

10 Bassuk, S. S. *et al.* Baseline characteristics of participants in the VITamin D and OmegA-3 TriaL (VITAL). *Contemp Clin Trials* **47**, 235-243, doi:10.1016/j.cct.2015.12.022 (2016).

11 Teng, E. L. & Chui, H. C. The Modified Mini-Mental State (3MS) examination. *J Clin Psychiatry* **48**, 314-318 (1987).

12 Evans, D. A., Grodstein, F., Loewenstein, D., Kaye, J. & Weintraub, S. Reducing case ascertainment costs in U.S. population studies of Alzheimer's disease, dementia, and cognitive impairment-Part 2. *Alzheimers Dement* **7**, 110-123, doi:10.1016/j.jalz.2010.11.008 (2011).

13 Rapp, S. R. *et al.* Validation of a cognitive assessment battery administered over the telephone. *J Am Geriatr Soc* **60**, 1616-1623, doi:10.1111/j.1532-5415.2012.04111.x (2012).

14 Altman, D. G. & Bland, J. M. Measurement in medicine: the analysis of method comparison studies. *Statistician* **32**, 307–317 (1983).

Supplementary Table S1

Table S1. Cognitive function at two assessments by vitamin D supplement assignment, for VITAL-Cog participants aged 60+ years, (n =3424) assessed by telephone and for CTSC-Cog participants aged 60+ years, (n=794) assessed in person: individual cognitive tests*

VITAL-COG (n=3424; telephone assessments)						CTSC-COG (n=794; in-person assessments)					
	Vitamin D Group		Placebo Group		Difference in score at each timepoint (Vitamin D–Placebo; 95% CI) ‡		Vitamin D Group		Placebo Group		Difference in score at each timepoint (Vitamin D–Placebo; 95% CI) ‡
	N	Mean (SE) ‡	N	Mean (SE) ‡			N	Mean (SE) ‡	N	Mean (SE) ‡	
Cognitive tests common to the two substudies†						Cognitive tests common to the two substudies†					
TICS 10 words - immediate recall					Difference in score‡	TICS 10 words immediate recall					Difference in score‡
1st assessment score	14	4.61 (0.04)	15	4.69 (0.04)	-0.08 (-0.20, 0.04)	1st assessment score	38	4.75 (0.07)	39	4.72 (0.07)	0.03 (-0.16, 0.22)
2nd assessment score	14	4.71 (0.04)	14	4.71 (0.04)	0.003 (-0.12, 0.12)	2nd assessment score	25	5.06 (0.08)	26	4.76 (0.08)	0.30 (0.06, 0.53)
TICS 10 words - delayed recall					Difference in score‡	TICS 10 words - delayed recall					Difference in score‡
1st assessment score	14	2.69 (0.05)	15	2.71 (0.05)	-0.02 (-0.15, 0.12)	1st assessment score	38	2.03 (0.09)	39	1.90 (0.08)	0.13 (-0.11, 0.37)
2nd assessment score	14	2.74 (0.05)	14	2.70 (0.05)	0.04 (-0.11, 0.18)	2nd assessment score	25	2.30 (0.11)	26	2.14 (0.10)	0.16 (-0.13, 0.46)
EBMT – immediate recall					Difference in score‡	EBMT – immediate recall					Difference in score‡
1st assessment score	14	9.59 (0.04)	15	9.59 (0.05)	0.003 (-0.12, 0.13)	1st assessment score	38	9.74 (0.09)	39	9.74 (0.08)	-0.001 (-0.23, 0.23)
2nd assessment score	14	9.57 (0.05)	14	9.51 (0.05)	0.07 (-0.07, 0.20)	2nd assessment score	25	9.94 (0.10)	26	10.05 (0.09)	-0.10 (-0.38, 0.17)
EBMT – delayed recall					Difference in score‡	EBMT – delayed recall					Difference in score‡
1st assessment score	14	9.23 (0.05)	15	9.23 (0.05)	-0.002 (-0.13, 0.13)	1st assessment score	38	9.34 (0.09)	39	9.32 (0.09)	0.03 (-0.21, 0.27)
2nd assessment score	14	9.15 (0.05)	14	9.16 (0.05)	-0.01 (-0.15, 0.14)	2nd assessment score	25	9.59 (0.10)	26	9.53 (0.10)	0.06 (-0.23, 0.34)
Animal naming test					Difference in score‡	Animal naming test					Difference in score‡
1st assessment score	14	19.24 (0.14)	15	19.57 (0.14)	-0.33 (-0.72, 0.06)	1st assessment score	38	21.01 (0.30)	39	20.29 (0.31)	0.73 (-0.11, 1.56)
2nd assessment score	14	19.09 (0.15)	14	19.36 (0.16)	-0.28 (-0.70, 0.15)	2nd assessment score	25	20.50 (0.33)	26	20.44 (0.32)	0.06 (-0.84, 0.97)

VITAL-COG (n=3424; telephone assessments)						CTSC-COG (n=794; in-person assessments)					
	Vitamin D Group		Placebo Group		Difference in score at each timepoint (Vitamin D–Placebo; 95% CI)[‡]		Vitamin D Group		Placebo Group		Difference in score at each timepoint (Vitamin D–Placebo; 95% CI)[‡]
	N	Mean (SE)[‡]	N	Mean (SE)[‡]			N	Mean (SE)[‡]	N	Mean (SE)[‡]	
Cognitive tests unique to each substudy[†]						Cognitive tests unique to each substudy[†]					
Digit span backwards					Difference in score[‡]	Vegetable naming test					Difference in score[‡]
1st assessment	14	6.73 (0.06)	15	6.83 (0.06)	-0.10 (-0.27, 0.07)	1st assessment	38	15.56 (0.23)	39	15.35 (0.22)	0.21 (-0.43, 0.85)
2nd assessment	14	1.97 (0.07)	14	2.04 (0.08)	-0.07 (-0.28, 0.13)	2nd assessment	25	15.19 (0.26)	26	15.13 (0.25)	0.06 (-0.65, 0.76)
Oral trails making test – Part A					Difference in score[‡]	Trails making test – Part A					Difference in score[‡]
1st assessment	14	10.66 (0.10)	15	10.27 (0.08)	0.39 (0.13, 0.65)	1st assessment	38	29.30 (0.58)	39	29.85 (0.47)	-0.55 (-2.02, 0.93)
2nd assessment	14	10.30 (0.14)	14	10.20 (0.14)	0.10 (-0.29, 0.49)	2nd assessment	25	28.40 (0.53)	26	30.59 (0.53)	-2.19 (-3.66, -0.73)
Oral trails making test – Part B					Difference in score[‡]	Trails making test – Part B					Difference in score[‡]
1st assessment	14	38.67 (0.62)	15	38.76 (0.62)	-0.09 (-1.82, 1.63)	1st assessment	38	80.15 (2.18)	39	82.78 (2.22)	-2.63 (-8.73, 3.47)
2nd assessment	14	39.26 (0.64)	14	38.64 (0.61)	0.62 (-1.10, 2.35)	2nd assessment	25	72.00 (1.89)	26	74.21 (1.97)	-2.21 (-7.57, 3.15)

Abbreviations: 3MS, Modified Mini-Mental Status exam (range=0-100);[11] CI, confidence interval; CTSC, Clinical and Translational Science Collaborative center for VITAL in Boston, MA; TICS, Telephone Interview of Cognitive Status (range=0-41)[1]

[*] **In the VITAL-Cog**, 2483 completed both assessments, 501 completed only the baseline, 440 completed only the 2nd assessment. **In the CTSC-Cog**, 497 completed both assessments, 279 completed only the baseline and 18 completed only the 2nd assessment.

[†] For a description of the tests, see previous section on “Supplemental Methods”.

[‡] Least squares mean and standard errors and differences of least squares means and standard errors were derived from univariate models.
